# TRIM44 Promotes Rabies Virus Replication by Autophagy-Dependent Mechanism

**DOI:** 10.3390/ijms25094616

**Published:** 2024-04-23

**Authors:** Hongling He, Ting Cai, Qiaozhu Chen, Zilian Chen, Boyue Zhang, Changyi Chen, Yueze Wang, Yan Liu, Yueming Wang, Yongwen Luo, Shile Huang, Jun Luo, Xiaofeng Guo

**Affiliations:** 1 College of Veterinary Medicine, South China Agricultural University, Guangzhou 510000, China; 20201028008@stu.scau.edu.cn (H.H.); 20213073005@stu.scau.edu.cn (T.C.); 20212028004@stu.scau.edu.cn (Q.C.); zlchen@stu.scau.edu.cn (Z.C.); zhangboyue163@163.com (B.Z.); archiechen2024@163.com (C.C.); wyz123@stu.scau.edu.cn (Y.W.); lyeah.scau.edu.cn@stu.scau.edu.cn (Y.L.); 18853858032@stu.scau.edu.cn (Y.W.); ywluo@scau.edu.cn (Y.L.); 2 Department of Biochemistry and Molecular Biology, Louisiana State University Health Sciences Center, 1501 Kings Highway, Shreveport, LA 71130-3932, USA; shile.huang@lsuhs.edu; 3 Department of Hematology and Oncology, Louisiana State University Health Sciences Center, 1501 Kings Highway, Shreveport, LA 71130-3932, USA; 4 Feist-Weiller Cancer Center, Louisiana State University Health Sciences Center, 1501 Kings Highway, Shreveport, LA 71130-3932, USA

**Keywords:** rabies virus, replication, E3 ubiquitin ligase, TRIM44, autophagy

## Abstract

Tripartite motif (TRIM) proteins are a multifunctional E3 ubiquitin ligase family that participates in various cellular processes. Recent studies have shown that TRIM proteins play important roles in regulating host–virus interactions through specific pathways, but their involvement in response to rabies virus (RABV) infection remains poorly understood. Here, we identified that several TRIM proteins are upregulated in mouse neuroblastoma cells (NA) after infection with the rabies virus using RNA-seq sequencing. Among them, TRIM44 was found to regulate RABV replication. This is supported by the observations that downregulation of TRIM44 inhibits RABV replication, while overexpression of TRIM44 promotes RABV replication. Mechanistically, TRIM44-induced RABV replication is brought about by activating autophagy, as inhibition of autophagy with 3-MA attenuates TRIM44-induced RABV replication. Additionally, we found that inhibition of autophagy with rapamycin reverses the TRIM44-knockdown-induced decrease in LC3B expression and autophagosome formation as well as RABV replication. The results suggest that TRIM44 promotes RABV replication by an autophagy-dependent mechanism. Our work identifies TRIM44 as a key host factor for RABV replication, and targeting TRIM44 expression may represent an effective therapeutic strategy.

## 1. Introduction

Rabies virus (RABV) belongs to the genus Lyssavirus in the family Rhabdoviridae. It is a single-stranded negative-sense RNA virus that causes acute inflammation of the nervous system in humans and other warm-blooded animals. Importantly, once clinical symptoms appear, the fatality rate is over 99.9% [1,2]. It is estimated that 59,000 people die from rabies worldwide each year [3]. The RABV genome sequence is approximately 12 kb in length and includes genes encoding the nucleoprotein (N), phosphoprotein (P), matrix protein (M), glycoprotein (G), and RNA-dependent RNA polymerase (L). The G protein is an important determinant of innate immune response and pathogenic mechanisms [4,5]. It is also a structural protein that can induce the production of virus-neutralizing antibodies (VNAs) in the host. The M and P proteins have been found to be associated with virus-induced autophagy [5,6]. Though there are numerous reports on the mechanisms of RABV infection and interactions with host factors [7], the specific pathways affected in host cells during RABV infection remain to be elucidated.

The tripartite motif (TRIM) protein family is a group of E3 ligases containing Really Interesting New Gene (RING) domains, which control important biological processes such as apoptosis, autophagy, signal transduction, innate immunity, and tumor development [8]. For example, downregulation of TRIM11 contributes to the pathogenesis of tau protein diseases, and restoration of TRIM11 expression may be an effective therapeutic strategy for Alzheimer’s disease [9]. TRIM21 is a key regulator of glycolysis in the body [10], and recent studies have shown that TRIM proteins also play critical roles in host immune responses or specific pathways involved in viral infections [11]. TRIM23 activates virus-induced autophagy through TANK-binding kinase 1 (TBK1) [12], TRIM7 promotes virus entry by ubiquitinating the envelope protein of the Zika virus [13,14], and TRIM25 inhibits the production of RABV through degradation of phosphoproteins and activation of RIG-1-mediated type I interferon [15]. Therefore, the involvement of TRIM proteins in viral infections is an emerging concept.

Autophagy is a major intracellular degradation system in eukaryotic cells that is precisely regulated by multiple signaling molecules [16]. It is known that various viruses can regulate autophagy for their replication [17,18,19]. RABV infection activates autophagy, thereby facilitating virus replication [5,20]. Many TRIM proteins, such as TRIM2, TRIM3, and TRIM71, are closely related to autophagy after viral infection [21]. It has been shown that TRIM44 interacts with the mitochondrial antiviral signaling protein (MAVS) to inhibit Sendai virus (SeV) replication [22] and negatively regulates interferon-induced promotion of viral replication in zebrafish infected with Singapore grouper iridovirus (SGIV) and red-spotted grouper nervous necrosis virus (RGNNV) [23]. Furthermore, it has also been suggested that the TRIM44 protein may serve as a bridge to link autophagy and proteasome degradation systems [24]. However, the role of TRIM44 in RABV infection and its effect on autophagy in host cells are still unclear. 

To further elucidate the mechanisms of RABV infection in host cells, we performed RNA-Seq transcriptome analysis of NA cells infected with RABV. We found that the expression levels of many TRIM proteins, including TRIM44, were upregulated. Interestingly, downregulation of TRIM44 expression reduced RABV replication, while overexpression of TRIM44 promoted RABV replication. Further research revealed that TRIM44 activated autophagy, thereby facilitating virus replication. This work provides new insights into the mechanism by which TRIM44 acts as a key host factor to promote RABV replication.

## 2. Results

### 2.1. RABV Infection Upregulates TRIM Proteins in NA Cells

**Figure 1:**
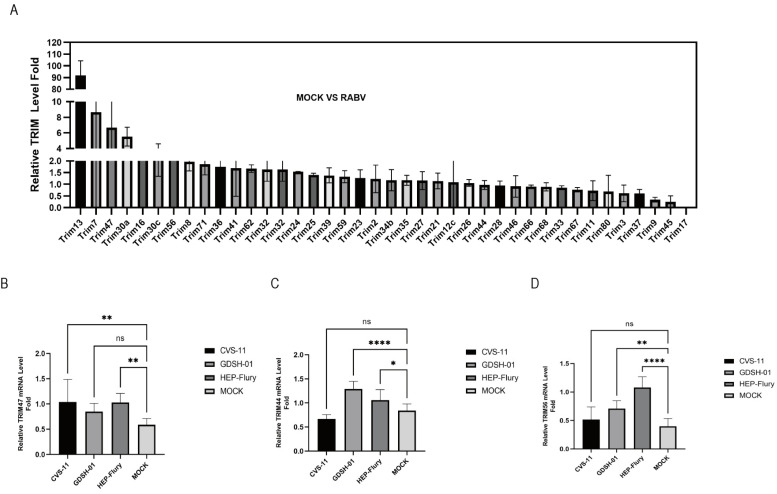
Upregulation of TRIM proteins in NA cells following RABV infection. (**A**) Transcript levels of TRIM proteins in NA cells infected with HEP-Flury for 24 h analyzed by RNA-seq. (**B**) The transcript levels of TRIM47, (**C**) TRIM44, and (**D**) TRIM56 proteins in NA cells infected with CVS-11, GDSH-01, and HEP-Flury (MOI = 1) for 24 h using RT-qPCR. Statistical analysis was performed using one-way ANOVA. ns, *p* > 0.05, * *p* < 0.05, ** *p* < 0.01, **** *p* < 0.0001.

To comprehensively investigate the potential host immune response to RABV, we performed RNA-seq analysis to identify the host factors involved in RABV infection. We found that various host factors, especially the TRIM protein family, were upregulated in NA cells after 24 h RABV infection (Figure 1A). Studies have shown that TRIM proteins play important roles in viral infections [25]. To confirm the impact of RABV infection on the expression of TRIM proteins, the mRNA levels of TRIM47, TRIM44, and TRIM56 were firstly determined using RT-qPCR after infection with different RABV strains. The results showed that the mRNA level of TRIM47 increased upon infection with CVS-11 and HEP-Flury strains (Figure 1B). The mRNA level of TRIM44 was upregulated upon infection with GDSH-01 and HEP-Flury strains (Figure 1C), and the mRNA level of TRIM56 was elevated upon infection with GDSH-01 and HEP-Flury strains (Figure 1D). These results suggest that RABV infection upregulates the expression of TRIM proteins in vitro.

### 2.2. Silencing of TRIM44 Inhibits RABV Replication

**Figure 2:**
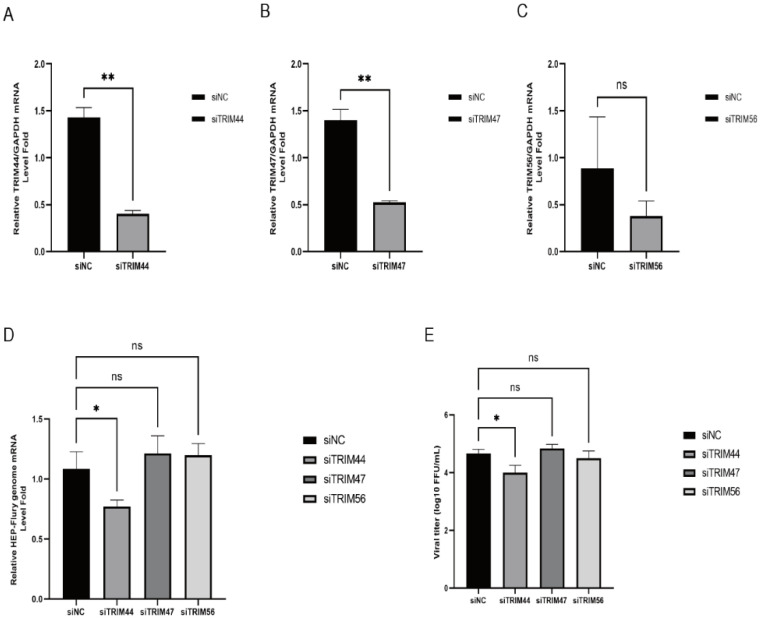
Silencing of TRIM44 inhibits RABV replication. NA cells were transfected with siRNA targeting TRIM44 (**A**), TRIM47 (**B**), or TRIM56 (**C**) for 24 h, followed by infection with RABV (HEP-Flury) at MOI = 1. Cells were collected at 24 h post-infection, and the transcript levels of TRIM proteins were analyzed by RT-qPCR. NA cells were transfected with siTRIM44 for 24 h, followed by infection with RABV (HEP-Flury) at MOI = 1. Cells were collected at 24 h post-infection, and RABV genome transcript levels were determined (**D**). Changes in RABV viral titers after the silencing of TRIM proteins (**E**). Statistical analysis was performed using a *t*-test or one-way ANOVA. ns, *p* > 0.05, * *p* < 0.05, ** *p* < 0.01.

To determine the impact of TRIM44, TRIM47, and TRIM56 proteins on RABV replication, corresponding siRNAs were used to silence TRIM44, TRIM47, and TRIM56. The transcript levels of the TRIMs and viral genome were examined using RT-qPCR, and virus titers were determined using a TCID50 assay. RT-qPCR results showed that siRNAs significantly reduced the transcript levels of TRIM44 (Figure 2A), TRIM47 (Figure 2B), and TRIM56 (Figure 2C). Analysis of viral genome transcription levels revealed that silencing TRIM47 or TRIM56 had no significant effect on the RABV viral genome. Interestingly, silencing TRIM44 significantly inhibited the RABV genome (Figure 2D) and virus titers (Figure 2E). These results indicate that the knockdown of TRIM44 affects RABV replication.

### 2.3. TRIM44 Is Crucial for Virus Replication

**Figure 3:**
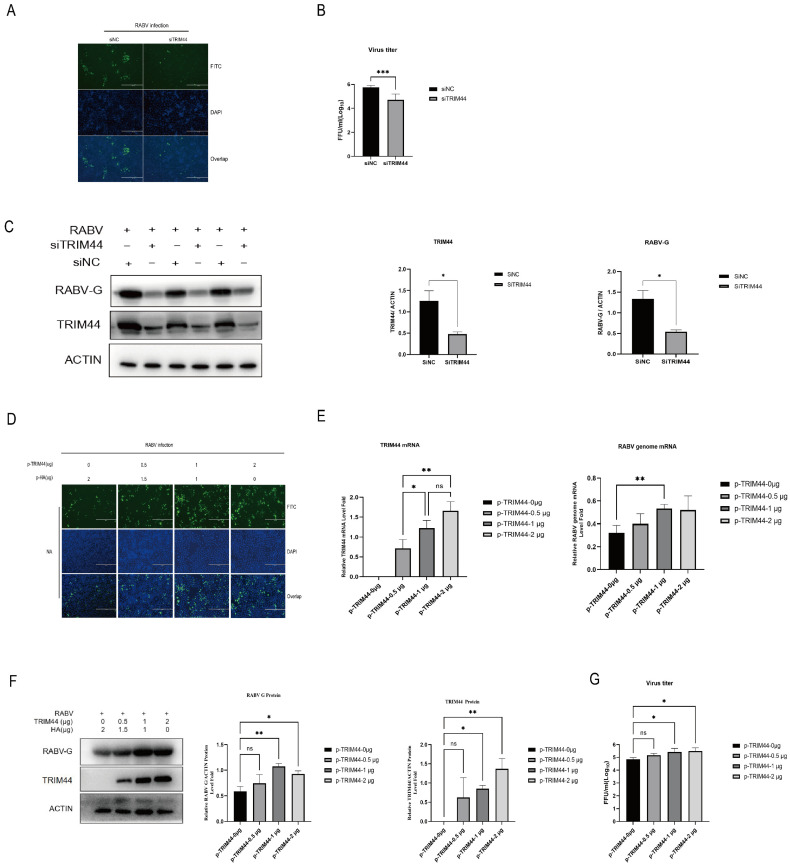
TRIM44 is crucial for virus replication. (**A**–**C**) NA cells were transfected with siTRIM44 to silence TRIM44 for 24 h, followed by infection with the RABV (HEP-Flury) virus (MOI = 1). Cells were collected at 24 h post-infection, and the expression of the RABV-N protein was directly detected by immunofluorescence staining, Bar = 400 μm (**A**). The viral titer was determined by TCID50 assay (**B**), and the levels of the RABV-G and TRIM44 proteins were analyzed by Western blotting (**C**). (**D**–**G**) NA cells were transfected with p-TRIM44 plasmid to overexpress TRIM44 for 24 h, followed by infection with the RABV (HEP-Flury) virus (MOI = 1). Cells were collected at 24 h post-infection, and the expression of the RABV-N protein was directly detected by immunofluorescence staining, Bar = 400 μm (**D**). The transcription levels of TRIM44 and the viral genome were determined by RT-qPCR (**E**), and the levels of RABV-G and TRIM44 proteins were analyzed by Western blotting (**F**). The viral titer was determined by TCID50 assay (**G**). Statistical analysis was performed using a two-tailed unpaired *t*-test or one-way ANOVA. ns, *p* > 0.05, * *p* < 0.05, ** *p* < 0.01, *** *p* < 0.001.

To further explore the role of TRIM44 in RABV infection, we investigated the effects of TRIM44 on RABV proliferation. Specific siRNA (siTRIM44) was used to knock down TRIM44 in NA cells, while pCAGGS-TRIM44-HA plasmid was used to transfect the cells to overexpress TRIM44. The cells transfected with siNC or pCAGGS-HA (empty vector) were used as corresponding controls. Immunofluorescence assay (IFA) results showed that depletion of TRIM44 in NA cells reduced the expression of RABV-N protein compared to the control group (Figure 3A). Consistent with the above observations, the knockdown of TRIM44 reduced the viral titer of RABV (Figure 3B) and RABV G protein expression (Figure 3C). In contrast, overexpression of TRIM44 increased the expression of RABV-N protein using IFA (Figure 3D). RT-qPCR results revealed that overexpression of TRIM44 increased the transcription of the RABV genome and G protein (Figure 3E). The level of RABV-G protein increased robustly after the overexpression of TRIM44, as detected by Western blotting (Figure 3F). Overexpression of TRIM44 showed higher viral titers compared with the control in the supernatants (Figure 3G). Taken together, these results indicate that TRIM44 protein promotes RABV replication and is crucial for the virus’s replication.

### 2.4. TRIM44 Promotes RABV Replication through an Autophagy-Dependent Mechanism

**Figure 4:**
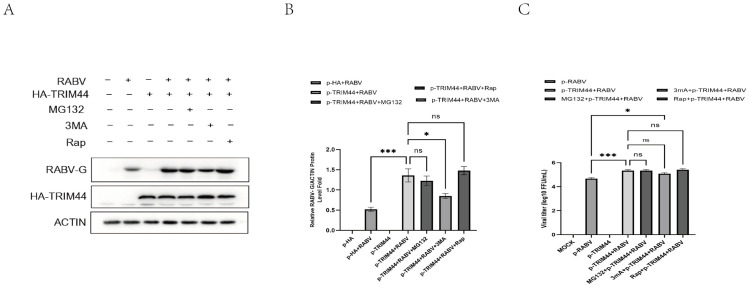
Autophagy inhibition attenuates TRIM44-mediated virus replication. NA cells were treated with DMSO, 3-MA (5 mM), rapamycin (Rap, 0.1 μM), and MG132 (1 μM), and then infected with RABV for 24 h at an MOI of 1.0. The relative expression of the RABV-G protein was evaluated by Western blotting (**A**). Statistical analysis of RABV-G protein bands in (**B**). The virus titers were determined by the TCID50 method (**C**). Statistical analysis was performed using one-way ANOVA. ns, *p* > 0.05, * *p* < 0.05, *** *p* < 0.001.

RABV infection activates autophagy, which facilitates the virus’s replication [21,22]. Also, it has been shown that TRIM21 promotes the production of RABV by ubiquitin-proteasome degradation of IRF7 [26]. To determine whether TRIM44 promotes RABV replication by activating the autophagy or proteasome pathway, Rap, (an autophagy inducer), 3-MA (an autophagy inhibitor), and MG132 (a proteasome inhibitor) were utilized. As shown in Figure 4A–C, overexpression of TRIM44 significantly increased the RABV-G protein levels and the virus titers in the RABV-infected cells compared to uninfected cells. The addition of 3-MA reversed the stimulatory effect of TRIM44 on RABV-G protein expression and RABV replication, whereas the addition of MG132 had no effect on the RABV-G protein levels and the virus titers elevated by TRIM44. The addition of Rap slightly enhanced TRIM44-induced expression of RABV-G, but failed to potentiate TRIM44-induced replication of the virus. These findings suggest that TRIM44 promotes RABV replication by activating the autophagy pathway rather than the proteasome pathway.

**Figure 5:**
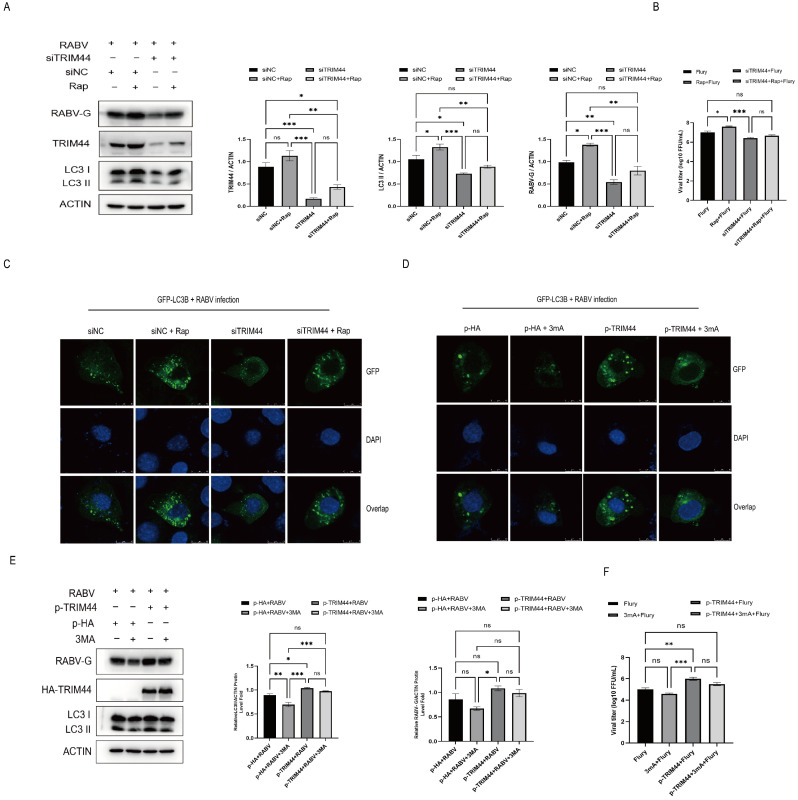
TRIM44 promotes RABV replication by activating autophagy. NA cells were transfected with p-TRIM44 or siTRIM44 for 24 h, treated with 3-MA (5 mM) or rapamycin (0.1 μM), and then infected with RABV for 24 h at an MOI of 1. The relative expression levels of the RABV-G, TRIM44, and LC3II proteins were determined by Western blotting (**A**,**B**). The formation of LC3II-positive puncta was observed by laser confocal microscopy, Bar = 10 μm (**C**,**D**). The virus titers were evaluated using the TCID50 method (**E**,**F**). Statistical analysis was performed using one-way ANOVA. ns, *p* > 0.05, * *p* < 0.05, ** *p* < 0.01, *** *p* < 0.001.

To substantiate that TRIM44 regulates RABV replication through an autophagy-dependent mechanism, NA cells were transfected with p-TRIM44 and/or siTRIM44 plasmids for 24 h, treated with 3-MA or Rap for 2 h, and then infected with RABV at an MOI of 1 for 24 h. The results showed that Rap treatment increased the levels of the RABV-G protein, LC3II protein (an autophagy marker), LC3-positive puncta (an indicator of autophagosomes), and virus titers compared to DMSO treatment. Rap treatment also restored the replication and autophagy inhibition caused by TRIM44 silencing (Figure 5A–C). Notably, 3-MA treatment significantly decreased the expression levels of the RABV-G protein and LC3II protein, and this inhibitory effect was reversed by TRIM44 overexpression (Figure 5D–F). Taken together, the results indicate that TRIM44 promotes RABV replication through an autophagy-dependent mechanism.

## 3. Discussion

The rabies virus (RABV) is an ancient neurotropic virus that can lurk in muscle tissues at the site of the wound, escape the immune response of the body, invade the central nervous system, and ultimately cause fatal encephalitis. Although we have some understanding of the overall picture of RABV, there are still many mysteries regarding the interaction between the virus and the host. Therefore, it is important to further study the potential mechanisms of RABV immune evasion in order to develop new effective therapeutic targets. TRIM family members are involved in many cellular functions, including immune system regulation, antiviral responses, autophagy-related receptor regulation, and cancer initiation [27]. Multiple studies have revealed the important role of TRIM family proteins in various viral replications. For example, it has been found that TRIM26 restricts the infection of EB virus in nasopharyngeal epithelial cells by connecting with HSP-90β through K48-linked ubiquitination [28]. Arterivirus nonstructural protein 1 can inhibit TRIM19 to promote virus replication [29]. Many TRIM proteins are also key factors in restricting autophagy regulatory pathways. Our previous studies have shown that TRIM21 promotes the production of RABV by ubiquitinating degradation of IRF7 [26], while TRIM25 inhibits the production of RABV by activating RIG-I-mediated type I interferon [15]. These studies indicate that TRIM proteins participate in viral infections by directly interacting with viral proteins or regulating specific pathways that are affected in the immune and host cells. In this study, we found that many TRIM proteins are significantly upregulated in RABV-infected NA cells through RNA-Seq. Autophagy plays an important role in RABV replication [30]. Studies have reported that TRIM44 not only participates in the innate immunity against viral infection [31] but also acts as a bridge between cellular autophagy and the ubiquitin–proteasome degradation system [24]. Similarly, TRIM47 is involved in the innate immunity against viral infection [32] and is associated with neuronal autophagy [33], while TRIM56 regulates replication of many viruses [34,35]. Therefore, we chose to explore TRIM44, TRIM47, and TRIM56, as these TRIM proteins’ mechanisms in RABV infection have not been studied. However, in the knockdown experiments, only TRIM44 knockdown affected RABV replication, and no significant difference was observed with TRIM47 and TRIM56 knockdown. Subsequently, we further investigated TRIM44 and found that overexpression of TRIM44 enhanced RABV replication. Clearly, the expression level of TRIM44 is positively correlated with RABV replication. Since its first cloning in 2001 [36], there have been few reports on the association of TRIM44 with viruses. Our work unveils the critical role of TRIM44 in RABV infection.

Although TRIM44 has been demonstrated to regulate viral replication through multiple pathways, the potential mechanism of TRIM44 in RABV infection has not been studied yet. During our study, we noticed that TRIM44 did not directly interact with RABV structural proteins, indicating that TRIM44 regulates RABV replication through other mechanisms. It is well known that RABV infection can activate autophagy, and autophagy can promote RABV replication [6,21]. Therefore, we speculated that TRIM44 may promote RABV replication through inducing autophagy. To test this hypothesis, we knocked out TRIM44 and treated cells with Rap (an autophagy inducer) or overexpressed TRIM44 and treated cells with 3-MA (an autophagy inhibitor). We found that knockdown of TRIM44 led to decreased LC3II expression, and the inhibition of RABV replication caused by TRIM44 silencing was restored by Rap treatment. On the other hand, overexpression of TRIM44 resulted in increased LC3II expression, and the stimulation of RABV replication triggered by TRIM44 overexpression was partly attenuated by 3-MA treatment. These findings suggest that TRIM44 promotes RABV proliferation only partly by enhancing autophagy. Further research is needed to identify other mechanisms by which TRIM44 regulates RABV infection.

In conclusion, here we found that TRIM44 is upregulated in NA cells during RABV infection. Overexpression and knockdown experiments revealed that TRIM44 has a promoting effect on RABV replication. Mechanistically, TRIM44 promotes RABV replication by activating autophagy. Overall, these findings reveal a crucial role of TRIM44 in RABV infection. This work enhances our understanding of the interplay between the host and the virus, providing a new strategy to combat rabies.

## 4. Materials and Methods

### 4.1. Reagents and Antibodies

The chemical reagents used in this study included 3-Methyladenine (3-MA, MedChemExpress, HY-19312, Shanghai, China), Rapamycin (Rap, Selleckchem, S1039, Shanghai, China), and (S,R,S)-(-)-MG-132 (MG132, Selleckchem, S2619, Shanghai, China), which were dissolved in dimethyl sulfoxide (DMSO) and stored at −80 °C. The primary antibodies used in this study were as follows: TRIM44 Polyclonal antibody (38KD) (Proteintech, 11511-1-AP, Chicago, IL, USA), LC3B (D11) Rabbit mAb (Cell Signaling Technology, 3868, Boston, MA, USA), β-ACTIN Rabbit mAb (ABclonal, AC026, Wuhan, China), mouse monoclonal anti-HA (Santa Cruz Biotechnology, sc-7392, Dallas, TX, USA). The secondary antibodies used for immunoblotting were horseradish peroxidase (HRP)-conjugated goat anti-mouse IgG (Beyotime, A0216, Shanghai, China) and HRP-conjugated goat anti-rabbit IgG (Beyotime, A0208, Shanghai, China). Mouse monoclonal anti-rabies virus glycoprotein (RABV-G, 58KD) antibody was prepared in the laboratory, and FITC-labeled anti-rabies virus nucleoprotein (RABV-N, 50KD) antibody was purchased from Fujirabio Diagnostics (Malvern, PA, USA).

### 4.2. Strains and Cells

The mouse neuroblastoma cell line (NA) (Wuhan Institute of Biological Products, Wuhan, China) was cultured in RPMI 1640 medium (Gibco, Oakland, CA, USA) supplemented with 10% fetal bovine serum (FBS) (Gibco). The fixed RABV HEP-Flury strain (gifted by Dr. Kongwang He from Jiangsu Academy of Agricultural Sciences, Nanjing, China) and RABV challenge virus standard 11 (CVS-11) strain (a gift from Dr. Xianzhu Xia, Academy of Military Medical Sciences, Beijing, China) were propagated in NA cells. The wild-type RABV strain GDSH-01 was isolated from the brain tissue of a pig infected with rabies by our laboratory [37].

### 4.3. RNA Sequencing (RNA-Seq)

NA cells were infected with or without RABV virus at an MOI of 1 and then harvested at 24 h post infection (p.i.) to analyze the host factors involved in the process of RABV infection. Total RNA in the cells was extracted with Trizol (Magen, Guangzhou, China) following the protocol of the supplier and used for RNA sequencing.

### 4.4. Plasmids and siRNA

The HA-tagged pCAGGS plasmid (pCAGGS-HA) was purchased from Miao Ling Biotechnology Co., Ltd. (Wuhan, China). The TRIM44 gene from NA cells was cloned into pCAGGS-HA using the primers TRIM44-F: 5′-ACCCATACGATGTTCCAGATTACGCTATGGCCTCCGGAGTGG-3′ and TRIM44-R: 5′-CCTTAATTAATTAAGATCTGCTAGCTCATGTGTCCTCTTCTTCACTGG-3′ for overexpression of TRIM44. The sequences of protein-targeting small interferon RNA (siRNA) and non-targeting control (NC) siRNA used in this study were listed in Table 1 and were synthesized by Sangon Biotechnology (Shanghai, China).

### 4.5. Virus Infection and Titer Determination

The NA cells were infected with RABV at a MOI = 1 when they reached 80% confluency. A control group was also set up. After 1 h incubation, the inoculum was removed and fresh culture medium was added, followed by incubation at 37 °C until harvest. The titer of RABV was determined by TCID50 assay. Briefly, the culture supernatants of cells infected with RABV were serially diluted 10-fold with RPMI 1640 medium. The diluted virus solution was then inoculated into NA cells in a 96-well plate and incubated at 37 °C for 48 h. Subsequently, direct immunofluorescence assay (IFA) was performed; the supernatant was discarded, and 80% acetone was added to fix the cells at −20 °C for 30 min. After fixation, the acetone was removed, and the cells were washed and incubated with FITC-labeled RABV nucleoprotein antibody at 37 °C for 1 h. The cells in the 96-well plate were observed under a fluorescence microscope (AMG, Washington, DC, USA), and the virus titer was calculated according to the Kaerber method.

### 4.6. Real-Time Fluorescence Quantitative PCR (RT-qPCR)

Cells were collected and total RNA was extracted using Trizol reagent. The total RNA was reverse transcribed (RT) using the TransScript First-Strand cDNA Synthesis SuperMix (Vazyme Biotech, Nanjing, China). Real-time quantitative PCR was performed on a CFX connect real-time system (Bio-Rad, Hercules, CA, USA) using SYBR Green Master Mix (Vazyme Biotech) according to the manufacturer’s instructions. The transcription levels of the target gene were normalized and calibrated to the level of the glyceraldehyde-3-phosphate dehydrogenase (GAPDH) gene. The primer sequences used are listed in Table 2.

### 4.7. Western Blotting

Cells were lysed with RIPA buffer (Beyotime, P0013B) supplemented with a protease and phosphatase inhibitor cocktail (Beyotime, P1045) and phenylmethanesulfonyl fluoride (PMSF, 1 mM). Protein concentration was determined using the Pierce™ BCA protein assay kit (Thermo Fisher Scientific, 23225, Waltham, MA, USA). Samples were separated by 10% or 12.5% SDS-PAGE and transferred onto polyvinylidene fluoride (PVDF) membranes (Millipore, ISEQ00010). After blocking with 5% skim milk at 37 °C for 1 h, the membranes were incubated with primary antibodies overnight at 4 °C, followed by incubation with HRP-conjugated secondary antibodies at 37 °C for 1 h. Protein bands were detected using the ECL Plus reagent kit (Beyotime, P0018) and imaged using the Tanon Fine Do X6 chemiluminescent imaging system (Bio-Equip, Shanghai, China). Target protein bands were semi-quantified using Image-Pro Plus 6.0 software. Density measurement results were reported as the ratio of the target protein to ACTIN.

### 4.8. siRNA and Plasmid DNA Transfection

NA cells were transfected with plasmid DNA or siRNA using Lipofectamine^®^ 3000 reagent (Thermo Fisher Scientific, L300015). Briefly, the appropriate amount of plasmid DNA or siRNA (with or without 2 μL P3000) was diluted in 50 μL serum-free OptiMEM (Thermo Fisher Scientific, 22,600,050), and 3 μL Lipofectamine^®^ 3000 was also diluted in 50 μL serum-free OptiMEM. Non-targeting control and empty plasmid were used as controls. The diluted solutions were mixed thoroughly and incubated at room temperature for 15 min. The mixture was then added to the cell culture plate and incubated at 37 °C for the specified time until harvest.

### 4.9. Laser Confocal Immunofluorescence Microscopy

NA cells were grown in 35-mm culture dishes with glass bottoms (Biosharp Life Science, BS-15-GJM, Hefei, China). After co-transfection with GFP-LC3 plasmid and pCAGGS-TRIM44 and/or pCAGGS-HA plasmids for 12 h, cells were treated with 3-MA or Rap for 2 h and then infected with RABV for 24 h. Live cells were washed twice with PBS and fixed with 4% paraformaldehyde (Sigma-Aldrich, P6148, Anseong, Republic of Korea) for 30 min. Cell permeabilization was performed with 0.2% Triton X-100 (Sigma-Aldrich, T8787) for 10 min, and cell nuclei were stained with DAPI (Beyotime, C1002). Images were acquired using a Leica confocal system (Leica TCS SP8, Weztlar, Germany).

### 4.10. Data Analysis

All data were expressed as mean values ± standard error (mean ± SE). All experiments were repeated three or more independent biological replicates. Data were analyzed using Graphpad Prism 9.5 software for statistical comparisons. Statistical significance was set at *p* < 0.05.

## Figures and Tables

**Table 1:** The sequences of protein-targeting siRNA and non-targeting control (NC) siRNA.

Gene	Sequence (5′~3′)
siTRIM44	F: GCAAUGAUAGAGUUGGUGGAATT
	R: UUCCACCAACUCUAUCAUUGCTT
siTRIM47	F: CUACAGAAACUCGGCUCAGAATT
	R: UUCUGAGCCGAGUUUCUGUAGTT
siTRIM56	F: CGAUAGAACCAAGAUAGGGAATT
	R: UUCCCUAUCUUGGUUCUAUCGTT
NC	F: UUC UCC GAA CGU GUC ACG UTT
	R: ACG UGA CAC GUU CGG AGA ATT

**Table 2:** The primers sequences used for RT-qPCR.

Gene	Sequence (5′~3′)
TRIM44	F:GCGGACATCCAATCTCACA
	R:TCGTCACCCTCTGCCTTT
TRIM47	F:GAGTTTCCAGAACGAGGTGAT
	R:TGCCGTGCCTTGCTTAG
TRIM56	F:GTTGACTTGGTGGGTTACAGAGC
	R:GAGAACAAGGTTGACAGAGGAAGC
GAPDH	F:CGTCCCGTAGACAAAATGGT
	R:TTGATGGCAACAATCTCCAC
HEP-Flury genome	F:AGAAGAAGCAGACATCGTCAGTTG
	R:GGAGACCACCTGATTATTGACTTTGA
HEP-G	F:GCCTTGATTGCCCTGATGTTGATAA
	R:CATTTCTCCCTGTCCCTCCAAGAT

## Data Availability

The authors confirm that the data supporting the findings of this study are available within the article.
